# Organic carbon generation in 3.5-billion-year-old basalt-hosted seafloor hydrothermal vent systems

**DOI:** 10.1126/sciadv.add7925

**Published:** 2023-02-01

**Authors:** Birger Rasmussen, Janet R. Muhling

**Affiliations:** School of Earth Sciences, The University of Western Australia, 35 Stirling Highway, Perth, WA 6009, Australia.

## Abstract

Carbon is the key element of life, and its origin in ancient sedimentary rocks is central to questions about the emergence and early evolution of life. The oldest well-preserved carbon occurs with fossil-like structures in 3.5-billion-year-old black chert. The carbonaceous matter, which is associated with hydrothermal chert-barite vent systems originating in underlying basaltic-komatiitic lavas, is thought to be derived from microbial life. Here, we show that 3.5-billion-year-old black chert vein systems from the Pilbara Craton, Australia contain abundant residues of migrated organic carbon. Using younger analogs, we argue that the black cherts formed during precipitation from silica-rich, carbon-bearing hydrothermal fluids in vein systems and vent-proximal seafloor sediments. Given the volcanic setting and lack of organic-rich sediments, we speculate that the vent-mound systems contain carbon derived from rock-powered organic synthesis in the underlying mafic-ultramafic lavas, providing a glimpse of a prebiotic world awash in terrestrial organic compounds.

## Teaser

Hydrothermal silica-carbon precipitation in 3.5 Ga basalt-hosted vent systems point to contribution from abiotic carbon synthesis.

## INTRODUCTION

**Fig. 1.**
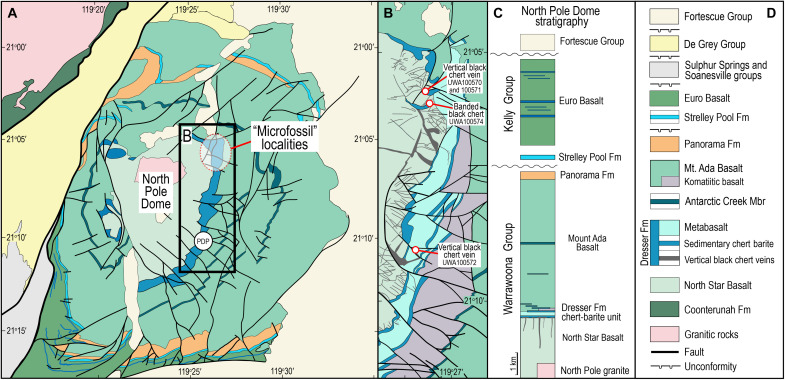
Locality map and stratigraphic column, North Pole Dome. (**A**) Simplified geological map of the North Pole Dome area. (**B**) Map of the eastern flank of the dome containing shallowly dipping chert and basalt transected by vertical black chert veins. (**C**) Simplified stratigraphic column. (**D**) Legend showing the stratigraphic units in the North Pole Dome area. “Microfossil” localities are from Dunlop *et al.* (*9*), Awramik *et al.* (*11*), and Ueno *et al.* (*4*). Fm, Formation; Mbr, Member; PDP, drill-hole Pilbara Drilling Project 2b.

The emergence of life on Earth required the formation of a diverse range of organic compounds. The contribution from terrestrial processes is unclear although outgassing and hydrothermal fluid-rock interactions are likely to have played important roles. Sedimentary rocks from Earth’s first billion years (Ga) have been strongly deformed and metamorphosed, making it difficult to determine whether the graphitic carbon they contain represents the remnants of microbial cells or the products of abiotic organic synthesis (*1*, *2*). The oldest well-preserved organic carbon occurs in ~3.5-Ga-old black bedded chert and chert veins in the basalt-dominated greenstone belt in the North Pole Dome, Pilbara Craton, Australia (Fig. 1). The carbonaceous matter [typically <0.2 weight % (wt%) total organic carbon] is dispersed in black chert, including in vertical veins several kilometers deep and several meters wide, which transect underlying basalts and terminate in a chert-barite unit, commonly forming synsedimentary barite mounds up to 15 m high and 50 m wide (*3*). The vertical vein-mound systems are interpreted to represent fossilized fluid conduits of hydrothermal vent systems (*3*–*7*).

The carbonaceous black cherts and intercalated sedimentary rocks contain purported microfossils and stromatolitic structures (*8*–*14*), as well as sulfur and carbon isotopic signatures (*4*, *5*, *15*–*17*), consistent with the establishment of life 3.5 Ga ago. However, the robustness of the various biosignatures has been questioned, including the biological origin of the ^13^C-depleted carbon (between −38.1 and −29.4‰) (*5*), which theoretically could have been produced by one or more abiotic processes (*18*, *19*). There is also uncertainty about whether the carbon in the black chert veins was indigenous, representing solid remains of microbial cells, or exotic, derived from particulate or dissolved organic material drawn down into vents or recharge zones (*20*, *21*). While most studies favor a biological origin for the carbon, the ubiquity of carbonaceous matter in hydrothermal chert veins originating deep in underlying mafic-ultramafic lavas has led some to invoke the possibility of a nonbiological origin (*22*–*24*).

In this study, we report results of in situ, nanometer-scale microscopy and microanalysis of ~3.5-Ga black cherts from the Dresser Formation in the North Pole Dome area (Fig. 1). Our results show that the chert veins contain abundant metamorphosed relicts of migrated organic compounds such as hydrocarbons. We argue that, using younger analogs, most of the carbon in the chert veins was originally an organic-rich liquid phase and that banded black chert around vertical veins formed by replacement of primary volcanogenic sediments by hydrothermal fluids enriched in dissolved silica and barium and organic-rich fluids. Our data support the proposal that carbonaceous matter in the black cherts is not de facto indigenous cellular carbon (i.e., kerogen), with implications for assessing the biogenicity of simple life-like microstructures. We conclude that the lack of kerogenous source rocks in the volcanic-dominated stratigraphy points to a contribution from abiotic organic compounds.

### Samples and geological setting

To investigate the origin of carbonaceous matter from the Dresser Formation, we prepared polished thin sections of chert from the North Pole Dome (*25*). The samples are from a 14-km N-S–oriented belt of chert on the eastern flank of the dome, which comprises shallowly dipping volcano sedimentary rocks of the Warrawoona Group intruded by a central monzogranite pluton (Fig. 1). Here, the group contains thick sequences of mainly tholeiitic basalt and lesser komatiite at its base (North Star Basalt) that are overlain by the Dresser Formation, which comprises five thin chert units intercalated with basalts (*3*). The lowermost chert (i.e., the “chert-barite” unit) is the thickest (≤60 m) and most extensive and preserves purported microfossils and stromatolites, as well as geochemical evidence for early biological activity.

The geological setting of the Dresser Formation is interpreted to be a submarine volcanic caldera (*3*, *7*), in which uplift during felsic volcanism led to faulting and synsedimentary hydrothermal activity with the development of chert-barite feeder veins in the underlying basaltic and komatiitic lavas. Cycles of magma injection potentially drove repeated episodes of uplift, extensional faulting and fracturing, and pulses of hydrothermal activity. The presence of structures interpreted to resemble modern geyserite, sinter terracettes, and mineralized remnants of hot spring pools suggests that the volcanic caldera and hydrothermal system were episodically emergent (*8*).

Trace element, fluid inclusion, and Si isotope data suggest that the vertical chert veins terminating in the Dresser Formation formed by mixing between deeply circulated surface fluids and magmatic volatiles (*26*, *27*). Fluid inclusions from thick quartz veins in the chert-barite unit indicate fluid temperatures between 300° and 120°C, whereas triple oxygen and Si isotope data for the Dresser cherts imply that microcrystalline silica precipitated from mixtures of seawater and hydrothermal fluids at temperatures of 150° to 170°C (*28*).

We also prepared polished thin sections of samples from the 340-million-year (Ma) Red Dog hydrothermal Zn-Pb-Ag-barite deposit, Alaska, USA, which comprises a series of stratabound sulfide deposits within siliceous, carbonaceous shale, chert, carbonate, and barite of the Mississippian Kuna Formation (*29*–*31*). The deposit is interpreted to have formed via a combination of sediment replacement and exhalation onto the seafloor. At Red Dog, early pervasive silicification of shale and carbonate preceded and coincided with hydrothermal massive sulfide mineralization. The widespread distribution of bitumen in the sulfide deposit and quartz veins (*31*) suggests that hydrocarbon generation and migration were synchronous with hydrothermal silicification and sulfide mineralization. The samples studied here are bioclastic carbonate conglomerates from the main pit. The conglomerates have undergone partial silicification and hydrocarbon infiltration, producing bitumen-stained chert and chalcedony, including carbonaceous filamentous microfossils around bitumen-stained fractures (*32*).

## RESULTS

### 3.5-Ga vertical black chert veins

**Fig. 2.**
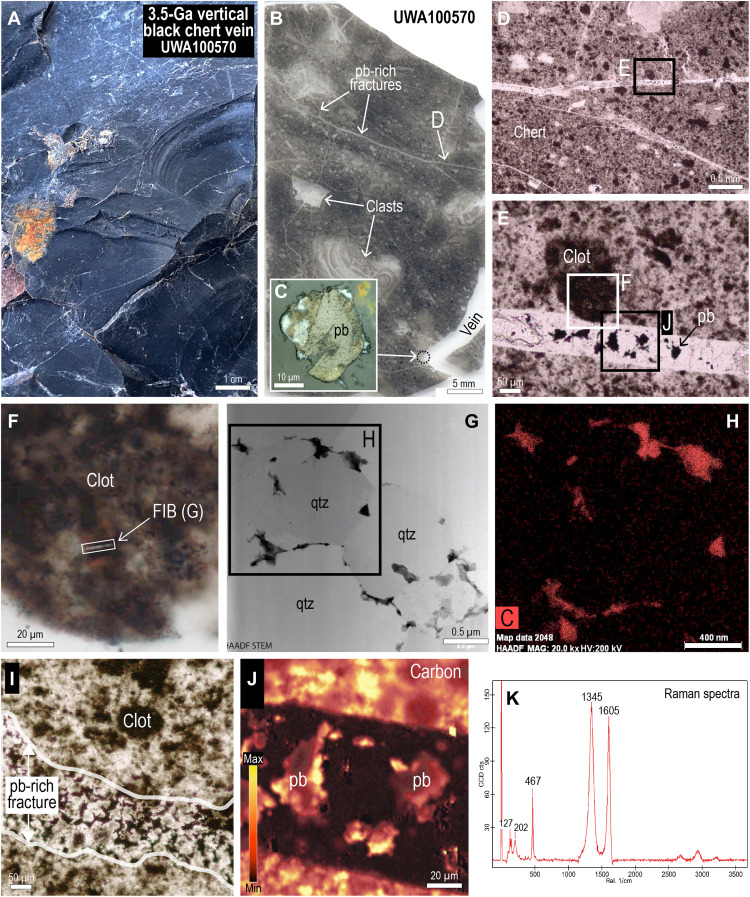
Carbonaceous matter in vertical black chert vein (UWA100570). (**A**) Image of vertical vein comprising black chert with conchoidal fracture. (**B**) Polished thin section of black chert with clasts and pyrobitumen (pb)–bearing fractures. (**C**) Reflected light (RL) image of pyrobitumen (pb) globule in cross-cutting chert vein. (**D** and **E**) Plane-polarized light (PPL) image of mottled black chert cross-cut by hairline fractures containing pyrobitumen (pb). (**F**) PPL image of clot showing the location of focused ion beam (FIB) pit. (**G**) High-angle annular dark-field (HAADF) scanning transmission electron microscopy (STEM) image of carbonaceous matter (black) coating quartz crystals (qtz) from FIB foil (F). (**H**) STEM-EDS carbon element map. MAG, magnification (**I**) PPL image of pyrobitumen (pb) in fracture cross-cutting mottled chert vein fill. (**J**) Raman map showing the distribution of carbon (acquired at its 1605-cm^−1^ band) from black chert transected by pyrobitumen-bearing (pb) fracture. (**K**) Raman spectra of carbonaceous chert showing two carbon peaks (the “D” disordered peak at 1345 cm^−1^ and the “G” graphite peak at 1605 cm^−1^), as well as quartz (peak at 467 cm^−1^). CCD, charge-coupled device.

Black chert veins terminating in the chert-barite unit comprise mostly microcrystalline quartz, with trace amounts of carbonaceous matter, pyrite, dolomite, iron oxides (after sulfides), and muscovite. The black chert veins contain angular clasts of silicified wall-rock, vein-filling silica and angular to rounded shapes of inclusion-free chert (Fig. 2). Fractures, veins, and microstylolites transect the chert and are commonly surrounded by haloes of gray bleached chert that contain less carbonaceous matter and pyrite reflecting partial oxidation.

**Fig. 3.**
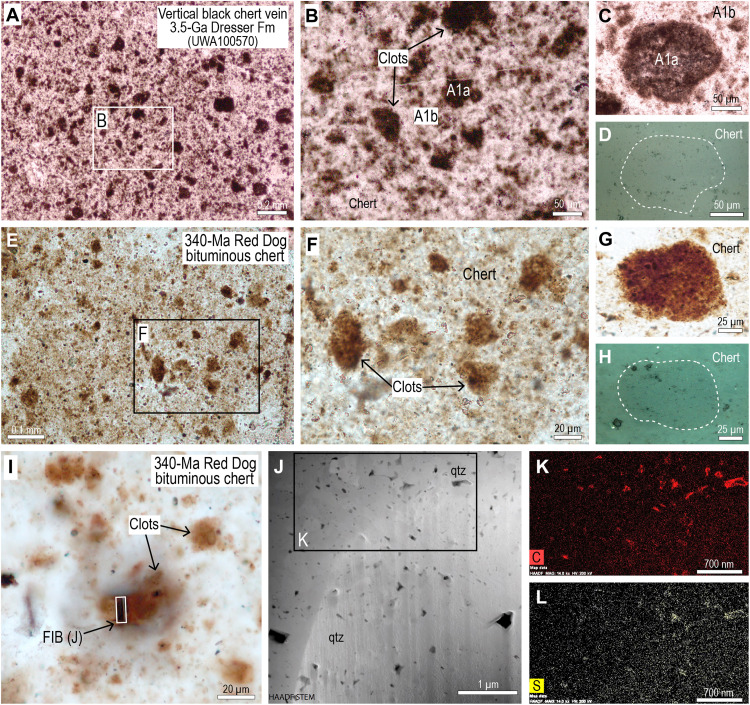
Comparison of carbonaceous clots in 3.5-Ga black chert veins with younger analog. (**A** and **B**) PPL images of mottled texture in 3.5-Ga black chert vein produced by darker clots (A1a) surrounded by lighter chert (A1b). (**C**) PPL image of semiopaque clot (A1a) surrounded by lighter chert (A1b). (**D**) RL image showing outline of clot in (C) comprising mostly chert with ultrafine carbonaceous matter. (**E** and **F**) PPL images of mottled brown chert from 340-Ma Red Dog conglomerate defined by irregular bituminous clots. (**G**) PPL image of large brown clot. (**H**) RL image showing outline of clot in (G) comprising mostly chert with ultrafine carbonaceous matter. (**I**) PPL image of brown bituminous clots in chert cement and FIB-pit. FIB, focused ion beam. (**J**) HAADF STEM image of FIB foil showing bitumen (black) between nanometer-sized quartz (qtz) crystals. (**K** and **L**) STEM-EDS element maps for carbon (K) and sulfur (L).

The chert that fills veins lacks sedimentary layering and has a mottled texture produced by darker subangular to rounded clots (mostly between 10 and 200 μm wide) evenly distributed in a lighter splotchy chert cement (Figs. 2 and 3), producing a pseudoclastic/breccia texture, characteristic of black chert veins in the Dresser Formation. In transmitted light, the clots are semiopaque to opaque with diffuse boundaries, whereas in reflected light, the opaque pigment is not resolvable.

**Fig. 4.**
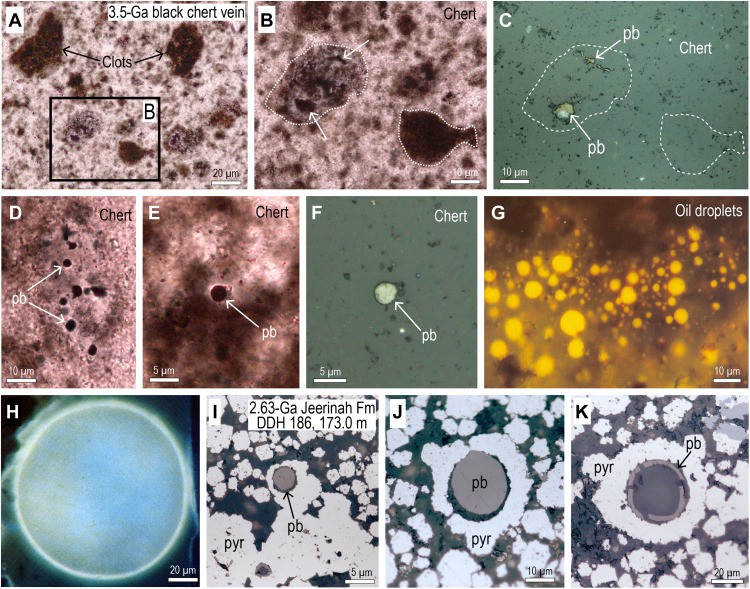
Pyrobitumen globules and fossilized oil droplets. (**A** and **B**) PPL images of carbonaceous clots in black chert vein. (**C**) RL image showing outline of clots in (B) that comprise mostly chert with larger masses of pyrobitumen (pb). (**D** and **E**) PPL images of pyrobitumen (pb) microspheroids in mottled black chert vein. (**F**) RL image of pyrobitumen (pb) microspheroid in (E). (**G**) Ultraviolet fluorescent light image of numerous small oil droplets (bright yellow) in matrix of organic-rich siltstone (cuttings, 14,320′ to 14,330′, Angola). (**H**) Image of isolated oil droplet in blue light (cuttings, 5750′, Papua New Guinea). (**I** and **J**) RL images of solid pyrobitumen (pb) microspheroids encased in diagenetic pyrite (pyr) cement from the 2.65-Ga Jeerinah Formation, drill-hole DDH186, 173 m. (**K**) Semihollow microspheroid comprising a thin layer of pyrobitumen (pb) with a quartz core surrounded by diagenetic pyrite (pyr). Fluorescent light images (G and H) from Alpern *et al.* (*40*), reproduced with permission from A. G. Dias, Universidade do Porto.

The carbon distribution in some of the clots is more heterogeneous, comprising areas of solid carbon of up to 2 μm in size (Fig. 4). The larger carbon aggregates are opaque in transmitted light and moderate to light gray-brown in reflected light (Fig. 4) and commonly are surrounded by an inner halo of carbon-free chert and an outer rim of finely dispersed carbonaceous matter, suggesting that they formed by coalescence of the finer carbon. Among the larger clots in the veins are minute (1 to 5 μm in diameter) spherical globules (Fig. 4, D to F) that closely resemble oil droplets from petroliferous shales (Fig. 4, G and H) and fossilized oil droplets in 2.65-Ga pyrite-cemented black shales (Fig. 4, I to K) (*33*). Similar microspheroids occur in carbonaceous laminae in banded black chert from elsewhere in the Dresser Formation (*34*).

To collect data on the nanometer-scale below the polished surface, we used a focused ion beam (FIB) to remove foils from dark clots in the black chert vein (Fig. 2) and pyrobitumen in narrow cross-cutting fractures. Transmission electron microscopy (TEM) imaging of the foil shows that the dark clots contain irregular, semicontinuous coatings of carbonaceous matter (<500 nm in width) concentrated along the surfaces of minute quartz crystals (<5 μm) (Fig. 2). Quartz crystals surrounded by carbon films tend to be smaller (0.1 to 1.0 μm) than adjacent quartz crystals (1 to 5 μm) (Fig. 2, G and H). TEM-energy dispersive X-ray spectroscopy (EDS) analysis shows that carbonaceous matter in the clots and pyrobitumen contain mainly C with minor S.

Raman spectrometry of the carbonaceous matter shows that it comprises highly disordered carbon with prominent peaks at 1345 cm^−1^ (D) and 1603 cm^−1^ (G), indicating temperatures of ~325°C using the full width half maximum of the G peak (*35*). These temperatures are consistent with previous estimates from carbonaceous matter in the Dresser Formation (~300°C) (*14*, *20*) and metamorphic mineral assemblages in Warrawoona Group basalts (*36*).

Carbonaceous matter interpreted to be pyrobitumen also occurs in veins (Fig. 2C) and narrow cross-cutting fractures (2 to 30 μm thick) that transect the mottled black chert (Fig. 2, D, E, and I). Most of the pyrobitumen is irregular in shape and occupies interstitial positions between quartz crystals. In reflected light, it is light to moderate gray-brown and has a speckled appearance. Raman spectrometry shows that the carbonaceous matter has a highly disordered structure and has been subjected to temperatures of up to 320°C, consistent with temperature estimates from carbonaceous clots in the adjacent chert (Fig. 2). TEM imaging of pyrobitumen shows that it occurs as interstitial masses between quartz crystals.

### Banded black chert, Dresser Formation

**Fig. 5.**
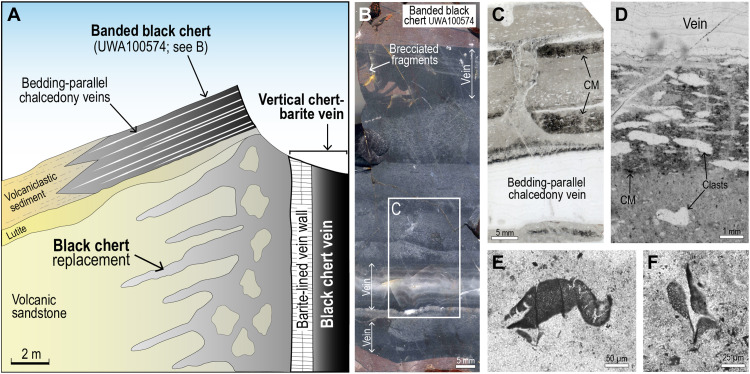
Primary volcanogenic sediment replaced by black chert (UWA100574). (**A**) Simplified cross section of outcrop showing replacement of primary sediments by black chert around vertical chert-barite vein [after (*39*)]. (**B**) Hand specimen of banded black chert comprising centimeter-thick clastic beds and bedding-parallel chert veins. (**C**) Image of polished thin section of banded black chert [see inset in (B)]. CM, carbonaceous matter. (**D**) PPL image showing outlines of silicified elongate grains in fine-grained matrix. CM, carbonaceous matter. (**E** and **F**) PPL images of volcanogenic clasts (dark brown) in silicified sediment.

Banded black chert from near-putative microfossils “Locality B” (*25*, *36*) comprises centimeter-thick beds with chert pseudomorphs of elongate clastic grains (up to 0.1 mm long) aligned with bedding (Fig. 5). The banded chert occurs within the silicification halo around a vertical hydrothermal black chert-barite vein (Fig. 5) (*25*, *34*). Although the sedimentary rock is composed almost entirely of chert, it preserves outlines of volcanic grains (Fig. 5, E and F), suggesting that the banded black chert was originally a fine- to coarse-grained volcanogenic sediment. The black pigment in the chert is patchily distributed and occurs as dark clots in a lighter speckled chert matrix (Fig. 5D).

**Fig. 6.**
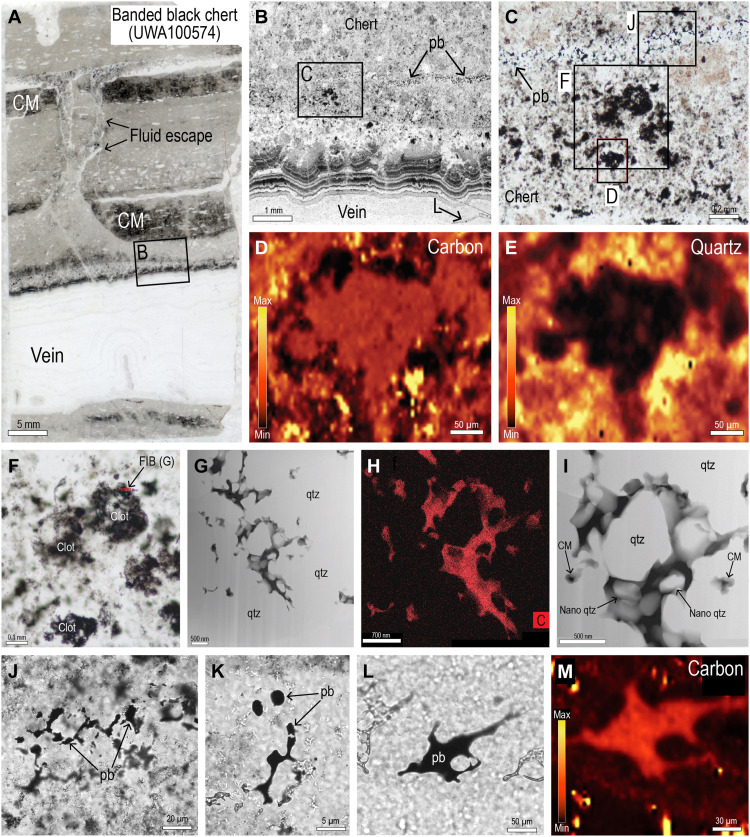
Carbonaceous matter in banded black chert. (**A**) Polished section of banded black chert with bedding-parallel vein of chalcedonic chert. (**B**) PPL image of silicified sediment and dark colloform chert along edge of vein. Pb, pyrobitumen. (**C**) PPL image of dark irregular clumps and thin fracture containing pyrobitumen (pb). (**D** and **E**) Raman maps showing finely disseminated carbon (D) in dark clot surrounded by fine-grained quartz (E). (**F**) PPL image of dark clot showing the location of FIB traverse. (**G** and **I**) HAADF STEM images of carbonaceous matter (CM, black) coating quartz (qtz) crystals from FIB foil. (**H**) STEM-EDS element map for carbon. (**J**) PPL image of interstitial pyrobitumen (pb) between quartz crystals in fracture. (**K** and **L**) PPL images of semispherical and smooth-surfaced pyrobitumen (pb) in fracture. (**M**) Raman map of globular pyrobitumen (L) showing distribution of carbon.

Raman spectrometry shows that the dark pigment in the clots is finely dispersed carbonaceous matter (Fig. 6, D and E). TEM observations from a FIB foil cut across the edge of a black clot show that the carbonaceous matter occurs as irregular, semicontinuous films concentrated along the edges of minute (<2 μm) quartz grains (Fig. 6). The carbonaceous matter encloses euhedral quartz crystals <300 nm long, whereas adjacent quartz crystals are embayed and locally enclose pockets of carbonaceous matter (Fig. 6, G to I). TEM-EDS analysis shows that the carbonaceous matter comprises mainly C, with minor S. The concentration of carbonaceous matter around quartz crystals (Fig. 6), which replaced primary volcanogenic sediments (Fig. 5), suggests that the carbon was deposited during or after silica precipitation.

The banded black chert is also cut by thin irregular fractures (5 to 20 μm wide) containing pyrobitumen that occurs in interstitial positions between microcrystalline quartz crystals (Fig. 6J) or as globular masses with smooth surfaces (Fig. 6, K to M). Raman spectrometry and mapping shows that the pyrobitumen in the fractures has been heated to ~330°C.

**Fig. 7.**
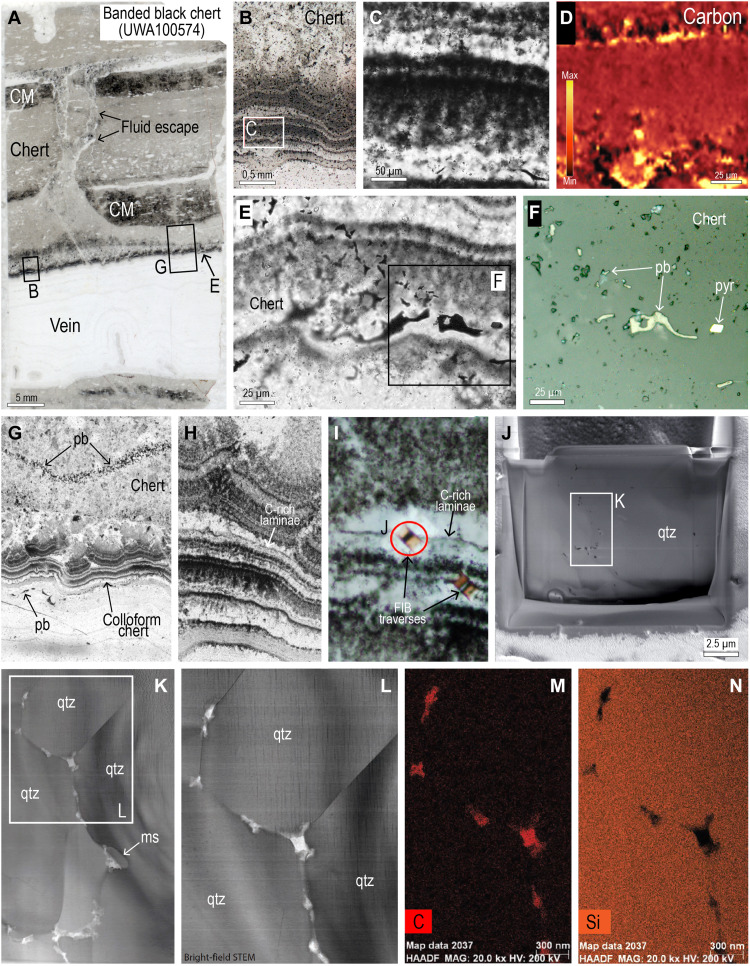
Pyrobitumen in colloform black chert. (**A**) Polished thin section of banded black chert with bedding-parallel vein of chalcedonic chert. (**B** and **C**) PPL images from edge of vein showing thin black and white laminae in dark chert. (**D**) Raman map showing finely dispersed carbonaceous matter in black chert. (**E**) PPL image showing pyrobitumen in colloform chert. (**F**) RL image of pyrobitumen (pb) in chert. Pyr, pyrite. (**G** to **I**) PPL images of black colloform chert showing the location of FIB traverses (I). FIB, focused ion beam. (**J**) Scanning electron microscope image of FIB-foil [see (I)]. (**K** and **L**) STEM bright-field images from FIB foil showing carbonaceous matter (white) and muscovite (ms) along quartz (qtz) grain boundaries and triple junctions. (**M** and **N**) STEM-EDS element maps for carbon (M) and silica (N).

The banded black chert is transected by several bedding-parallel veins (1 to 2 cm thick) comprising colloform bands of chert, including bands of radially extinguishing chert that is pseudomorphous after fibrous chalcedony. Thinly laminated colloform chert along the edge of the vein is stained black by a dark pigment identified by Raman spectrometry as carbonaceous matter (Fig. 7). The dark colloform chert is locally transected by thin fractures containing pyrobitumen (Fig. 7, E and F). Nanometer-scale TEM observations of a FIB foil from the colloform black chert shows that the carbonaceous matter is concentrated along quartz crystal boundaries, particularly at triple junctions (Fig. 7, K to N).

### Bituminous chert in 340-Ma Red Dog Zn-Pb-barite deposit, Alaska

In samples of bioclastic carbonate conglomerate from the Red Dog mine, bitumen is associated with postdepositional chert and chalcedony, indicating that the sediments were infiltrated by liquid hydrocarbons during postdepositional silicification (*32*). A characteristic feature of the secondary chert, which fills cavities and has replaced the edges of carbonate grains, is the presence of a mottled texture defined by irregular, brown clots (up to 100 μm) comprising thin residues of carbonaceous matter between fine-grained quartz crystals (Fig. 3, E to H). TEM observations from a FIB foil removed from a brown clot (Fig. 3I) show that it contains carbonaceous matter between nanoquartz crystals (Fig. 3J). TEM-EDS element maps show that the carbonaceous matter is composed mainly of C, with minor S (Fig. 3, K and L), consistent with the composition of bitumen in the same sample (*32*). The clots, which formed during the codeposition of fine-grained silica and migrated hydrocarbons, closely resemble the carbonaceous clots in the 3.5-Ga black chert veins from the Dresser Formation (Fig. 3, A to D), suggesting a similar mode of formation.

## DISCUSSION

### Origin of carbonaceous matter in banded black chert

Carbonaceous matter in Precambrian cherts, such as the 1.88-Ga stromatolitic black cherts of the Gunflint Formation, Ontario, Canada, has traditionally been interpreted to be kerogen, that is, the thermally altered remains of indigenous cellular carbon (*37*). However, recent studies show that at least some of the carbonaceous matter was derived from migrated hydrocarbons that infiltrated the chert and were solidified and altered to pyrobitumen during burial and subsequent metamorphism (*38*). Differentiating kerogen from pyrobitumen in Precambrian sedimentary rocks hinges largely on the habit and morphology of the carbonaceous matter (*39*, *40*). In contrast to kerogen (*40*–*42*), which is largely derived from depositional organic matter, pyrobitumen was formerly a fluid and therefore tends to occupy cavities, fractures, and veins; coat surfaces; and form discrete droplets (*38*–*40*).

The ~3.5-Ga black chert veins and banded black chert-barite unit contain carbonaceous matter that occupies fractures and cavities (Figs. 2 and 7) and is, therefore, interpreted to be pyrobitumen. Likewise, finely dispersed carbonaceous matter in bedding-parallel veins (Fig. 7, B to D) filled by banded chalcedony and quartz is likely to be migrated carbon as documented in chalcedony from the Red Dog deposit. Solid carbonaceous microspheres in chert bands and veins (Fig. 4, D to F) most closely resemble fossilized oil droplets (Fig. 4, I to K).

The origin of the irregular dark clots (Figs. 2 to 4) is less clear. The increase in carbon content in chert adjacent to the hydrothermal vents (Fig. 5), which has also been observed in younger black cherts (*43*), suggests that the distribution of the carbon is related to the hydrothermal fluids. Although the possibility has been raised that the C-rich clots may have formed by adhesion of organic compounds in the hydrothermal fluids, it is generally thought that the clots formed by in situ growth, possibly by chemotrophic microbial colonies or by adhesion of microbial colonies and/or extracellular polymeric substances on particles settling though the water column (*43*). However, these processes cannot readily explain the presence of dark clots in black chert veins deep below the paleosurface.

### Hydrothermal silica and carbon precipitation in black chert veins

Several lines of evidence suggest that the dark clots in the vertical chert veins formed from migrated organic compounds, including (i) their association with small globular masses resembling solidified oil droplets (Fig. 4), (ii) the occurrence of solid carbonaceous matter in putative fluid inclusions (*44*), (iii) the presence of carbonaceous clots in late-stage chert veinlets cross-cutting vein-filling mottled black chert (*3*), and (iv) the close similarity of the 3.5-Ga clots to brown bituminous clots in chert from Red Dog Zn-Pb deposit (Fig. 3), which formed during synchronous hydrothermal silicification and hydrocarbon migration.

Additional evidence for hydrocarbon migration in the Dresser Formation comes from the presence of semispherical masses of carbonaceous matter [figure 2D in (*13*)] resembling bitumen nodules, which form by radiation-induced solidification of organic fluids around U- and/or Th-rich minerals (*33*, *45*). In addition, possible oil droplets have been reported in a boulder of banded chert from a breccia lens in the chert-barite unit (*45*). More broadly, there is evidence for the presence of migrated carbon in other Paleoarchean cherts, such as the Apex chert where carbon in chert veins has infiltrated adjacent wall rocks (*21*). In black chert from the top of the Onverwacht Group, South Africa, cross-cutting veinlets contain carbonaceous matter, indicating the migration of organic compounds (*46*).

A migrated origin for the carbonaceous matter helps to explain its puzzling occurrence in cross-cutting chert veins and fissures, some originating up to 2 km below the paleosurface in underlying basalts and komatiites. The close association of carbonaceous matter and chert suggests that both phases were deposited from hydrothermal aqueous fluids carrying liquid and gaseous organic compounds. The precipitation of amorphous silica was probably triggered by a sharp drop in silica solubility associated with the ascent of hydrothermal fluids in the vertical vein systems.

**Fig. 8.**
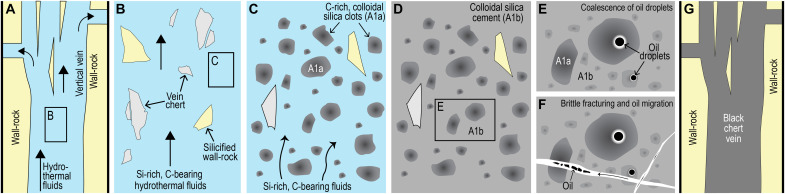
Diagram depicting the origin of black chert veins. (**A**) Development of vertical vein systems during extensional faulting and migration of hydrothermal Si-rich aqueous fluids and organic-rich phase. Episodic overpressure and hydraulic fracturing linked to silicification of fluid conduits on or below paleoseafloor. (**B**) Brecciation of silicified wall-rock and vein-filling silica. (**C**) Rapid precipitation of carbon-rich silica clots (A1a) from hydrothermal aqueous and organic fluids. Organic compounds coat and/or fill interstitial positions within amorphous silica phase. (**D**) Precipitation of C-bearing silica cement (A1b) and sealing of the veins. (**E**) Coalescence of finely dispersed liquid organic compounds into larger globules and droplets. (**F**) Transformation of amorphous silica into fine-grained quartz followed by multiple generations of brittle fracturing and organic carbon migration. (**G**) The formation of vertical black chert veins and horizontal fissures.

The earliest and most abundant material in the vertical veins is dark, rounded to angular C-rich clots which “float” in a lighter, speckled chert cement [Figs. 2 to 4; fabric “A1” in (*22*)]. We suggest that the dark clots were among the earliest vein-filling material, comprising nanometer-sized silica coated with carbon (termed here A1a), which precipitated from hydrothermal solutions enriched in dissolved silica and organic compounds (Fig. 8). The coalescence of finely dispersed carbon into larger globules within the clots (Figs. 4 and 8E) suggests that the carbon compounds were fluid. A subsequent influx of silica-rich solutions in the vein resulted in the precipitation of interstitial carbon-bearing silica cement (termed here A1b), producing the characteristic pseudoclastic/breccia fabric in black chert veins (A1). As with the black chert veins from the Apex chert (*22*), the Dresser veins have undergone multiple episodes of fracturing, silica cementation, and hydrocarbon migration (Fig. 8F).

The development of breccias, brittle fractures, and bedding-parallel fissures in and around the black chert vent system has been documented elsewhere and indicates hydraulic fracturing (*47*, *48*). Overpressure conditions may have developed following the silicification of overlying seafloor sediments, forcing Si-, Ba-, and carbon-bearing hydrothermal fluids into unlithified subseafloor sediments adjacent to fluid conduits. This process is indicated by the lateral transition from noncarbonaceous volcanogenic sediments into stratiform black chert around vertical black chert veins (Fig. 5) (*25*). It follows that banded black cherts are the products of subseafloor replacement of primary volcanogenic sediments by carbon-bearing, silica-rich hydrothermal fluids. A postdepositional origin for some of the carbonaceous matter in banded black chert implies that biological carbon production in the water column was lower than currently inferred.

### Source of hydrocarbons in 3.5-Ga black chert

#### Biological sources

**Fig. 9.**
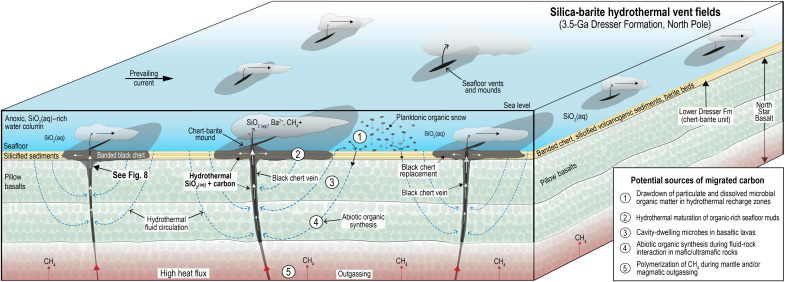
Generalized model of seafloor hydrothermal vent field 3.5 Ga ago. Hydrothermal fluid circulation through basaltic and komatiitic lavas driven by shallow magma and high heat flow. Potential sources of organic carbon generation include thermal maturation of microbial carbon (1 to 3), reduction of CO_2_ during serpentinization and carbonation reactions (4), and mantle outgassing (5). Codeposition of silica and organic compounds during ascent and cooling of hydrothermal fluids produced black chert veins and replaced volcanogenic sediments around seafloor vents. After (*3*, *4*, *6*, *20*).

The identification of pyrobitumen in the black chert veins and hydrothermally silicified sediments from the Dresser Formation (Figs. 2 to 4, 6, and 7) raises the question as to how the migrated organic compounds formed (Fig. 9). The conversion of biological organic matter, mostly derived from photosynthetically fixed carbon, to petroleum typically occurs when organic-rich mudrocks undergo heating through the “oil window” (60° to 150°C) during burial (*49*). However, petroleum generation also occurs in modern submarine hydrothermal systems, where ascending hot fluids cause near-instantaneous hydrous pyrolysis of immature organic matter on or below the seafloor (*50*, *51*). In the Guaymas Basin in the Gulf of California, numerous hydrothermal mounds (up to 20 to 30 m high and ~50 m wide) located above the rifted seafloor are stained and cemented with petroleum (*50*, *51*). The hydrocarbons, which were extracted from samples comprising massive sulfides, barite, and other hydrothermal minerals, are composed of aliphatic and aromatic hydrocarbons as well as polar asphalitic material (*51*). Whereas the volatile component (C_1_-C_10_ hydrocarbons) is dispersed upon emission into the water column, the heavier hydrocarbons (C_10_-C_40_+) solidify and are deposited in fluid conduits and cavities between the hydrothermal minerals (*50*, *51*).

Unlike the hydrothermal mounds from the Guaymas Basin, which overlie ~300 m of immature organic-rich sediments with an average organic carbon content of ~2 wt% (*50*), the chert-barite mounds in the Dresser Formation overlie thick sequences of basaltic and komatiitic lava. The lack of organic-rich sediments in the Dresser Formation and underlying basalts (*4*, *5*) argues against hydrothermal alteration of microbial biomass as a major source of migrated organic carbon (Fig. 9). Although sedimentary black cherts surround the vent systems, they contain only trace amounts of organic matter (typically <0.1 wt%) (*4*).

Another possible carbon source may have existed in the fractures, crevices, and vesicles in underlying lavas (Fig. 9), which in younger basalts are known to host a vast subseafloor ecosystem of chemoautotrophic and heterotrophic microorganisms (*52*–*54*). However, today, the subseafloor microbial biosphere is estimated to account for only 0.18 to 3.6% of total living biomass on Earth (*55*) and is an area of very low biological productivity that preserves little or no organic matter. In addition, there is currently no evidence for microfossils or carbonaceous matter in the basalts above and below the Dresser Formation.

Given the lack of organic-rich source rocks, some have proposed that the carbonaceous matter was derived from seawater particulate and dissolved microbial organic matter in seawater that was drawn down into the basaltic-komatiitic rock pile in recharge zones (Fig. 9) during hydrothermal circulation and deposited as kerogen in the chert veins (*20*). A similar process has been invoked for the origin of carbonaceous matter in black chert from the nearby and slightly younger (~3.46 Ga) Apex chert (*21*). However, the presence of a water column with particulate organic matter should result in the accumulation of high–total organic carbon muds, particularly in anoxic seawater, and the deposition of organic compounds in the underlying lavas during hydrothermal circulation. The lack of organic-rich mudrocks or reduced carbon in the basaltic lavas argues against this process as a major source of carbon in the black chert veins.

#### Rock-powered organic synthesis

The widespread distribution of reduced carbon in veins originating in basaltic and komatiitic lavas deep below the chert-barite unit points to a potential contribution from abiotic hydrocarbons (Fig. 9). Although the ^13^C-depleted isotopic composition of carbonaceous matter in black chert from the North Pole area (δ^13^C, −38.1 to −29.4‰) (*4*, *5*, *20*) is consistent with biological fractionation, laboratory experiments and analysis of Martian meteorites show that abiotic processes can also produce reduced organic compounds depleted in ^13^C (*19*, *56*, *57*). While more work is needed to investigate abiotic carbon synthesis and C isotope fractionation (*19*), the presence of ^13^C-depleted carbonaceous matter, particularly in Earth’s oldest rocks, does not uniquely indicate a biological origin.

Two of the main sources of abiotic hydrocarbons in the crust include outgassing from the upper mantle and in situ generation via reduction of inorganic carbon in the crust (*19*). Although the concentration of hydrocarbons emitted from modern igneous-dominated vent systems is small (*50*), higher heat flow and H_2_ production on the early Earth may have sustained a larger flux of deep-sourced reduced gases such as CH_4_ and other hydrocarbon gases, which were emitted at seafloor vents and eruption centers (*58*). Theoretical, experimental, and field observations also suggest that abiotic hydrocarbons form during fluid-rock interactions in the crust, leading to the production of H_2_ and the reduction of CO_2_ (*18*, *19*). Abiotic synthesis has been proposed for hydrocarbons in highly reducing environments such as alkaline submarine vents above ultramafic rocks undergoing serpentinization (*59*–*61*) and in fractures in Precambrian basement rocks (*62*). The formation of carbonaceous matter in Paleoarchean cherts has been tentatively linked to hydrothermal fluid-rock interactions, including (i) the reduction of CO_2_ via Fischer-Tropsch–type synthesis in veins (*6*, *22*), (ii) the thermal decomposition of Fe-bearing carbonate in banded chert beds (*23*), or (iii) serpentinization reactions producing H_2_ and reducing CO_2_ to CH_4_ during circulation of surface water through ultramafic rocks (*24*).

Although more work is needed to determine how the carbon in the 3.5-Ga chert vein system formed, its occurrence in deep-seated veins in mafic-ultramafic lavas and the absence of organic-rich source rocks implies a contribution from nonbiological sources. Hydrothermal fluid-rock interactions between seawater and a wide range of Fe(II)-rich igneous minerals and carbonate (*63*–*65*) may have produced a broad range of organic compounds on the prebiotic Earth. Direct petrographic evidence for the fluid-rock reactions producing abiotic carbon in ancient mafic-ultramafic lavas is likely to have been overprinted or destroyed on Earth, but evidence may exist on Mars where well-preserved volcanic rocks of >3.5 Ga old are exposed on the planet’s surface. Martian meteorite (ALH 84001) preserves nanotextural evidence consistent with abiotic synthesis of organic compounds during serpentinization and carbonation reactions between aqueous fluids and orthopyroxene (*65*).

### Implications for early Earth

Our results indicate that black chert in vertical vein-vent systems formed from silica and organic carbon deposited from hydrothermal fluids circulating through submarine mafic-ultramafic lavas. These findings represent a major advance toward understanding these common yet enigmatic structures, and the puzzling occurrence of carbonaceous matter in the deep-seated plumbing systems of seafloor hydrothermal vents.

The migration and deposition of carbon compounds from hydrothermal solutions in Earth’s oldest well-preserved volcano sedimentary rocks may have ramifications in the search for biosignatures. Carbonaceous matter in Paleoarchean black chert is generally interpreted to be derived from indigenous cellular carbon. Its presence in simple filaments and spheres is considered to be a key factor in assessing the biogenicity of putative fossil-like objects (*12*, *66*–*68*). Our results show that some of the carbonaceous matter in the 3.5-Ga cherts is not indigenous but exotic and, because of the lack of organic-rich source rocks, may include abiotically produced carbon. The migration of fluid hydrocarbons through veins, fractures, and permeable sediments around seafloor vent systems, coating mineral surfaces and filling cavities, could have produced a variety of carbonaceous microstructures, including possible organic biomorphs (*23*, *69*), which may be mistaken for microfossils.

In the context of prebiotic chemistry, our findings suggest that a proportion of the organic compounds in the black chert veins may have formed via abiotic reduction of inorganic carbon during hydrothermal fluid-rock interactions in mafic-ultramafic igneous rocks and mantle outgassing. Before the evolution of life, the formation of organic compounds in hydrothermally active, volcanic-dominated settings may have acted as a continuous source of locally derived building blocks for biosynthesis, including chains of *n*-alkanes, which are a key component of fatty acids that are widely considered to have formed the first protocell membranes (*70*, *71*). The flux of organic compounds from Earth’s interior into the primitive oceans and lakes may have maintained a transiently more reducing hydrosphere conducive to biosynthesis around vent systems and seeps below a nonreducing N_2_-CO_2_ atmosphere. Given the abundance of black chert vent systems in Paleoarchean basalts from the North Pole area, the volume of low–molecular weight organic compounds emitted into the water column may have been substantial , affecting the chemistry of the early ocean and atmosphere and mitigating the effects of a faint young sun.

## MATERIALS AND METHODS

### Optical microscopy

Polished thin sections (~30 μm thick) were prepared and examined by an optical and scanning electron microscope. Routine optical microscopy, using transmitted and reflected plane-polarized light, was carried out on polished thin sections to collect mineralogical and textural information about the samples from the North Pole area and the Red Dog Zn-Pb deposit.

### Raman spectrometry

Raman spectrometry of carbonaceous matter in the chert beds and veins was carried out at the Centre for Microscopy, Characterisation, and Analysis (CMCA), University of Western Australia (UWA) using a WITec alpha 300RA + microscope and Control Five software, combined with a Peltier-cooled 1024-pixel by 1280-pixel charge-coupled device detector and a WITec 532 laser source. A 20×/0.5 objective was used for laser focusing. Spectra were acquired in the 0- to 3500-cm^−1^ range with spectral grating (600 liters/mm) calibrated against the 520-cm^−1^ band of pure silicon. The acquisition time was 15 s with 10 accumulations. Project Five software (WITec GmbH) was used for background correction and generation of Raman maps.

### Focused ion beam

Lamellae for TEM analyses were cut from polished thin sections of black chert veins and beds from the Dresser Formation (North Pole Dome, Western Australia) and from brown bituminous chert from the Kuna Formation (Red Dog Zn-Pb deposit, Alaska). FIB techniques were used to prepare ~100-nm-thick TEM lamellae using an FEI Helios NanoLab G3 CX DualBeam instrument located at CMCA, UWA. The areas selected for TEM analysis were first coated with a strip of Pt, 2 μm thick to protect the surface, and then trenches 7 μm deep were milled on either side of the strip using a Ga ion beam with 30-kV voltage and 9.3-nA current. The lamellae were then cut away from the samples and welded to Cu TEM grids. The lamellae were thinned with the Ga ion beam at 30 kV and 0.79 and 0.23 nA, before cleaning at 5 kV and 41 pA and polishing at 2 kV and 23 pA.

### Transmission electron microscopy

TEM data were obtained at 200 kV using an FEI Titan G2 80–200 TEM/scanning TEM (STEM) with ChemiSTEM technology located at CMCA, UWA. Bright-field TEM and STEM, high-resolution TEM, and high-angle annular dark-field STEM images were collected and processed using TIA (TEM Imaging and Analysis) software from FEI. Qualitative EDS spectra and maps were collected with an FEI Super-X EDS detector and processed using Esprit software from Bruker Corporation.
